# Effects of lockdowns on neurobiological and psychometric parameters in unipolar depression during the COVID-19 pandemic

**DOI:** 10.1038/s41398-024-02733-1

**Published:** 2024-01-19

**Authors:** Jakob Unterholzner, Alexander Kautzky, Murray Bruce Reed, Theresa Friederike Wechsler, Valentin Popper, Benjamin Spurny-Dworak, Peter Stöhrmann, Manfred Klöbl, Nimmy Varghese, Andreas Mühlberger, Anne Eckert, Richard Frey, Dan Rujescu, Rupert Lanzenberger, Thomas Vanicek

**Affiliations:** 1 Department of Psychiatry and Psychotherapy, Medical University of Vienna, Vienna, Austria; 2 Comprehensive Center for Clinical Neurosciences and Mental Health, Medical University of Vienna, Vienna, Austria; 3 Department for Psychology, Clinical Psychology and Psychotherapy, University of Regensburg, Regensburg, Germany; 4 Neurobiology Lab for Brain Aging and Mental Health, Transfaculty Research Platform Molecular & Cognitive Neuroscience (MCN), University of Basel, Basel, Switzerland

**Keywords:** Depression, Molecular neuroscience

## Abstract

Defying the COVID-19 pandemic required restriction measures of unprecedented scale, that may induce and exacerbate psychiatric symptoms across the population. We aimed to assess in vivo dynamic effects of mitigation strategies on human brain neurobiology, neuroplastic as well as psychometric parameters. Three structural magnetic resonance imaging measurements, serum brain-derived neurotrophic factor (sBDNF) analyses, and psychometric assessments (Beck Depression Inventory-II and Perceived Stress Questionnaire-20) were performed in healthy individuals and patients with a recurrent major depressive disorder in the period from September 2020 to July 2021. Group differences and changes over time in structural imaging, neuroplastic and psychometric parameters were assessed with linear mixed models. Analysis of data from 18 patients with a recurrent major depressive disorder and 28 healthy individuals showed clinically relevant scores for depression and stress in the patient group as well as significant cross-sectional differences in depression scores (F = 30.89, *p* < 0.001) and three subscales of the Perceived Stress Questionnaire (Worries: F = 19.19, *p* < 0.001, Tension: F = 34.44, *p* < 0.001, Joy: F = 12.05, *p* = 0.001). Linear mixed models revealed no significant changes over time in cortical thickness of the prefrontal cortex, anterior cingulate cortex, hippocampus, and amygdala (F = 0.29, *p* > 0.1) and no interaction with group (F = 0.28, *p* > 0.1). Further, analysis revealed no main effect of time and no interaction of time x group in depressive symptoms, perceived stress subscales, and sBDNF (all *p* > 0.1). Despite the limited sample size, the strength of this investigation lies in the multimodal assessment of peri-pandemic lockdown effects. Nine months of varying restrictions measures did not result in observable changes in brain morphology nor impact depressive symptoms in either psychiatric patients with a recurrent major depressive disorder or healthy individuals. While these neurobiological and psychometric data stand in contrast to initial expectations about the effects of restriction measures, they might inform future investigations of longitudinal effects of restriction measures on mental health.

## Introduction

The COVID-19 pandemic brought along extensive restrictions on daily living, which were implemented to reduce the spreading of the Sars-CoV-2 virus. Globally, these mitigation strategies were enforced in varying degrees of severity for the infected and contact persons but also for non-exposed, non-infected individuals. Data from previous outbreaks of contagious diseases suggest that mental health symptoms are common during outbreaks [1] and that quarantine results in long-lasting psychological impact depending on various factors [2]. Regarding the increasing prevalence of stress, anxiety, and depression among the general population during the initial phase of the COVID-19 pandemic, a surge of post-traumatic stress disorder was expected, especially when considering the unprecedented scale of the restrictions during the COVID-19 pandemic [3]. Perceived stress during the lockdown was shown to be associated with the development of depressive symptoms in the general population [4]. Individuals with a mental disorder might exhibit a higher vulnerability for external stressors due to gene*environment interactions, as suggested by the diathesis-stress model [5] and newer concepts such as the differential susceptibility framework [6]. Interestingly, however, changes in psychological symptoms during the COVID-19 pandemic seemed less apparent for the population with pre-pandemic mental disorders [7]. In addition to psychometric parameters, changes in brain function, structure and neuroplasticity constitute further adaptational aspects that are involved in the maintenance of mental well-being in response to external stressors [8]. Currently, the literature on neurobiological mechanisms that influence coping strategies and the development of psychiatric symptoms and syndromes in response to restriction measures is sparse. Changes in brain structure have been reported in response to traumatic life events [9, 10]. An observational study demonstrated decreased volume of the dentate gyrus, the parahippocampal gyrus, the dorsolateral prefrontal cortex and the orbitofrontal cortex, after 14 months of social isolation [11]. These reductions in grey matter volume were associated with an attenuation of brain-derived neurotrophic factor (BDNF) levels. BDNF is highly expressed in hippocampal regions and frequently discussed as a peripheral marker of neuroplasticity, learning and physical activity [12].

With the implementation of lockdown and contact reduction measures, the COVID-19 pandemic presents a natural, real-world setting allowing to monitor longitudinal changes in different levels of adaptational processes during a stressful life event. For this purpose, we recruited subjects that had already participated in studies at our department, including patients with a recurrent major depressive disorder (MDD) and subjects without any known mental, neurological, or internal disorders. Based on previous findings we hypothesised that there would be a cumulative effect of lockdown and social restriction measures on the volumes of the amygdala, hippocampus, and of the anterior cingulate cortex and prefrontal cortex, a reduction in serum BDNF levels in both groups and increasing levels of perceived stress and depressive symptoms over time. Furthermore, we expected a stronger reduction in brain grey matter, and stronger increases in perceived stress and depressive symptoms in individuals with recurrent MDD in comparison to healthy participants.

## Material and methods

### Population

**Fig. 1:**
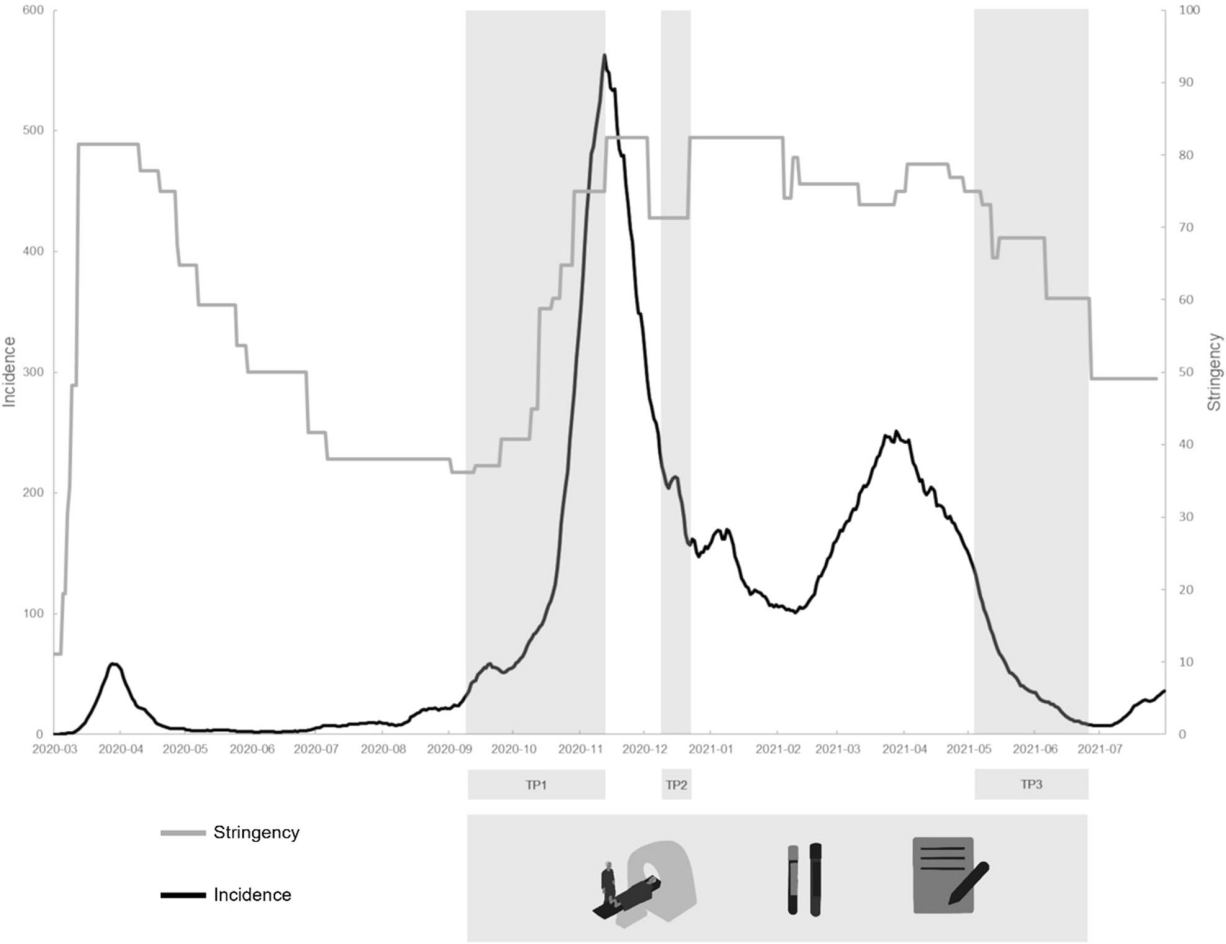
Timeframe of the study with respective measurement phases (TP1-TP3, light grey), including MRI, blood draw and psychometric tests. TP – time point. (The COVID19 stringency index is composed of nine response metrics, including travel bans and stay-at-home requirements, to assess the strictness of government measures; 0-100 (lowest to highest).).

The sample population of the current study comprised subjects that had participated in previous studies at our department (clinicaltrials.gov Identifier: NCT02810717 and NCT02753738; ethics committee numbers: EK 1761/2015 and 1739/2016). Individuals were excluded in case of a suspected or confirmed SARS-CoV-2 infection, severe concomitant somatic/neurological disorder (or any mental disorder for healthy subjects) that had developed since the previous study participation, any implant or stainless-steel graft serving, a contraindication for MRI measurements, or a positive pregnancy test or breast-feeding. The baseline examination included a Structured Clinical Interview for DSM-IV (SCID-IV), as well as a physical and blood examination. After inclusion, subjects were measured three times (MRI, blood draw, psychometry) during the Covid19-pandemic from September 2020 until July 2021 (shown in Fig. 1). In total, 30 healthy individuals and 21 patients with a recurrent MDD were screened to be included in this investigation. One individually previously included in the group of healthy individuals showed depressive symptoms at the first study visit and one only completed the first MRI measurement. Two participants with a known recurrent MDD showed an ongoing remission, thus not fulfilling the criteria for a depressive episode and were thus not included in the analysis, another individual with an MDD did not fulfil MRI inclusion criteria. Within this quasi-experimental and naturalistic study, treatment was introduced or adapted according to individual needs and based on clinical indication and shared decision making of patients and clinicians. Psychopharmacological treatment was changed in ten patients during the study period. None of the patients were treated in an in-patient setting or received ECT or TMS treatment over the course of the study. Six patients continued to receive an out-patient psychotherapy. For a detailed overview on patient characteristics see Table 1.

**Table 1:** Patient characteristics.

ID	Main diagnosis	Comorbid psychiatric disorders	Medication change during study	Medication at time point 1	Medication at time point 2	Medication at time point 3	Current psychotherapy	Previous treatment with	
								ECT	Ketamine
1	F33.2		yes	Lamotrigine 100 mg	n.a.	n.a.		x	x
2	F33.1	F41.0, F40.2, F90.0	no	Quetiapine 25 mg	Quetiapine 25 mg	Quetiapine 25 mg	x		x
3	F33.2	F60.6, F60.7	yes	Paroxetine 30 mg	Paroxetine 25 mg	Escitalopram 15 mg, Pregabalin 150 mg	x		
4	F33.1		no	n.a.	n.a.	n.a.	x		
5	F33.1		no	Clomipramine 75 mg, Buspirone 10 mg	Clomipramine 75 mg, Buspirone 10 mg	Clomipramine 75 mg, Buspirone 10 mg	x		
6	F33.1		no	Sertraline 125 mg, Duloxetine 60 mg	Sertraline 125 mg, Duloxetine 60 mg	Sertraline 125 mg, Duloxetine 60 mg			
7	F33.1	F90.0, F60.3	yes	Escitalopram 20, Methylphenidate 20 mg, Zolpidem 5 mg	Escitalopram 20 mg, Methylphenidate 20 mg, Aripiprazol 3 mg, Valdoxan 25 mg	Escitalopram 20 mg, Methylphenidate 10 mg, Aripiprazole 4 mg, Zolpidem 10 mg			
8	F33.1		no	Zoldem 10 mg, Chlorprotixene 100 mg, Tranylcypromine 40 mg	Zoldem 10 mg, Chlorprotixene 100 mg, Tranylcypromine 40 mg	Zoldem 10 mg, Chlorprotixene 100 mg, Tranylcypromine 40 mg		x	x
9	F33.3	F43.1	yes	Levomepromazine 100 mg, Lithium 675 mg, Tranylcypromine 45 mg, Bupropion 450 mg	Tranylcypromine 30 mg, Bupropion 150 mg, Prothipendyl 160 mg, Aripiprazole 5 mg, Lithium 450 mg	Tranylcypromine 30 mg, Bupropion 150 mg, Prothipendyl 160 mg, Aripiprazole 5 mg, Lithium 450 mg	x	x	x
10	F33.1		yes	n.a.	n.a.	Venlafaxine 300 mg, Mirtazapine 45 mg, Esketamine nasalspray (once/week)			
11	F33.1		yes	n.a.	Trazodone 100 mg	Bupropion 300 mg, Trazodone 150 mg			
12	F33.1		yes	Venlafaxine 150 mg, Mirtazapine 30 mg	Venlafaxine 150 mg, Trazodone 50 mg	Venlafaxine 150 mg, Trazodone 50 mg			
13	F33.1	F90.0	yes	n.a.	Methylphenidate 30 mg, Aripiprazole 3 mg, Quetiapine 25 mg	Methylphenidate 40 mg, Quetiapine 25 mg			
14	F33.1		no	Milnacipran 50 mg, Pegabalin 300 mg, Trazodone 100	Milnacipran 100 mg, Pregabalin 350 mg, Trazodone 150 mg	Milnacipran 100 mg, Pregabalin 350 mg, Trazodone 150 mg	x		
15	F33.1	F61	yes	n.a.	n.a.	Venlafaxine 300 mg, Lamotrigine 100 mg			x
16	F33.2		no	Bupropion 600 mg, Flupentixol/Melitracen, Milnacipran 50 mg	Bupropion 600 mg, Flupentixol/Melitracen, Milnacipran 50 mg	Bupropion 600 mg, Flupentixol/Melitracen, Milnacipran 50 mg			
17	F33.1	F90.0, F60.3	yes	n.a.	n.a.	Venlafaxine 75 mg, Qeutiapine 25 mg, Methylphenidate 30 mg			
18	F33.1		no	Sertraline 50 mg, Bupropion 150 mg, Lorazepam 1 mg	Sertraline 50 mg, Bupropion 150 mg, Lorazepam 1 mg	Sertraline 50 mg, Bupropion 150 mg, Lorazepam 1 mg		x	x

*ECT* electroconvulsive therapy

The authors assert that all procedures contributing to this work comply with the ethical standards of the relevant national and institutional committees on human experimentation and with the Helsinki Declaration of 1975, as revised in 2008. All procedures involving human subjects/patients were approved by the Ethics Committee of the Medical University of Vienna and the General Hospital of Vienna (EC number: 1410/2020). Written informed consent was obtained from all subjects.

### Timeframe and context

#### Measures in Austria during the COVID-19 pandemic (September 2020 until June 2021)

The first case of COVID-19 was reported on 25^th^ of February 2020. Similar to other countries worldwide, Austria responded to the increasing numbers of COVID-19 infections with measures to contain the further spread of the virus, with the first lockdown beginning on 16^th^ of March 2020. All businesses selling non-essential goods were closed, supermarkets and pharmacies remained open. Measures were eased and adjusted starting from 14^th^ of April 2020. The next restrictions were imposed on 3^rd^ of November with curfew, and another ‘hard’ lockdown on 17^th^ of November 2020 lasting until 7^th^ of December 2020. Another lockdown followed from 26^th^ of December 2020 until 08^th^ of February 2021. From 1^st^ of April until 19^th^ of May another lockdown followed.

The first hard lockdown (16^th^ of March 2020) as well as the light and hard November lockdown (3^rd^ and 17^th^ of November 2020) were applied nationwide. The April (or ‘Easter’) lockdown (1^st^ of April until 19^th^ of May) only concerned eastern Austria including Vienna, Lower Austria and Burgenland. The respective opening phases or the loosening of restriction measures were also applied on a nationwide basis, with small fluctuations according to regional differences in incidence rates. The study population comprised individuals living in Vienna and Lower Austria, two neighbouring Austrian states with great similarities in the imposed lockdowns and restriction measures.

Within the timeframe of the COVID-19 pandemic, the study visits correspond with the respective ‘loosening’ phases of government-imposed restriction measures. The first phase of measurements was conducted from 9^th^ of September until 12^th^ of November 2020, the second phase from 9^th^ until 22^nd^ of December 2020 (one subject was measured on the 29^th^ of December 202) and the third phase from 05^th^ of May until 10^th^ of June 2021 (shown in Fig. 1).

### Clinical assessment and online questionnaires

Mental symptoms and psychological distress as well as socioeconomic and psychosocial factors were collected using an online platform (EvaSys V8.0, Electric Paper Evaluationssysteme GmbH). Depressive symptoms were measured using the Beck Depression Inventory-II (BDI-II), referring to the last two weeks. Perceived stress was assessed using the German modified version of the Perceived-Stress-Questionnaire (PSQ-20), comprising the subscales worries, tension, joy, and demands, all referring to the last four weeks. The online questionnaires were performed at each visit (i.e., the three MRI measurements).

### Image acquisition and data processing

MRI measurements were performed on a 3 Tesla MAGNETOM PRISMA scanner (Siemens Medical, Erlangen, Germany) at the excellence centre for highfield MRI (MedUniWien, University Clinic for Radiology and Image Guided Therapy). Subjects were scanned using a 64-channel head coil. Structural MRI was performed using a magnetisation-prepared rapid gradient echo (MPRAGE) T1-weighted sequence (TE = 1800 ms, TR = 2.37 ms, 208 slices, 288×288 matrix size, slice thickness 0.85 mm, in-plane resolution 1.15 × 1.15 mm). An automated recon-all pipeline in FreeSurfer 7.1 software with default parameters was used for cortical surface reconstruction and parcellation of 34 cortical regions in each hemisphere and of subcortical regions (Harvard Medical School, Boston, USA; http://surfer.nmr.mgh.harvard.edu/) according to previous morphological imaging studies [13, 14]. Data of all time points were used to create a within-participant template via inverse consistent registration for the longitudinal processing pipeline. All volumes were visually inspected to maintain high quality segmentations. Total cortical volume and surface area were corrected for estimated intracranial total volume (eTIV). Cortical thickness scales with head size only to a small extent. Correcting for intracranial volume would thus add noise and provide inaccurate data and hence, when investigating cortical thickness correction for eTIV is not recommended [15, 16].

### Serum BDNF sampling and assessment

Blood samples were drawn using serum vacutainer tubes (Becton Dickinson) and a sodium citrate tube. After 30 min at room temperature the serum tube was centrifuged at 1500 × *g* for 15 min. Samples were stored at -80 °C until further use. Serum BDNF levels were assessed with an enzyme-linked immunosorbent assay (ELISA) kit (Biosensis® Mature BDNF Rapid™ ELISA Kit: Human, Mouse, Rat; Thebarton, SA, Australia). Samples were appropriately diluted (1:100 or higher if necessary) and detection of BDNF was carried out on a pre-coated mouse monoclonal anti-mature BDNF 96-well plate as described in the manufacturer’s protocol. Within five minutes after addition of stop solution, absorbance was measured in a microplate reader set at 450 nm and a correction wavelength set to 690 nm to determine BDNF concentrations according to the standard curve. The assays were performed in duplicate and the mean of both were calculated. For analysis of intrinsic assay quality, intra- and inter-assay coefficients of variation (CV) were assessed.

### Statistical analysis

We performed linear mixed-model analyses to assess the effects of multiple lockdowns on brain measures, sBDNF, and psychometric scores. According to the hypotheses, we performed a linear mixed model on the volumes of the amygdala, hippocampus, and cortical thickness and surface area of the anterior cingulate cortex and prefrontal cortex. Subsequently, we performed an explorative analysis, including data from all cortical and subcortical brain areas. Models were computed respectively for cortical thickness, surface area, and subcortical volumes as dependent variables. For each model, time point (factor, 3-levelled), group (binomial, patients with an MDD and HI), hemisphere (binomial, left and right), and ROI (factor) were included as fixed effects, and subject as the random intercept. Interaction effects were included up to four-way-interactions between group, time, hemisphere, and ROI and excluded instances of non-significance (starting from the highest interaction level). The potential confounding factors age, sex, and medication (factorial, 3 levels: HI, no changes of medication, changes of medication during the study period) were accounted for as fixed effects. Then, we carried out linear mixed models for BDI, PSQ-20 sub-scores, and sBDNF levels as outcome variables, time point and group as fixed factors, subject as the random intercept, with age, sex, and medication (see above) as covariates. Further, we performed Pearson correlations between sBDNF values and hippocampus and amygdala volumes, and cortical thickness of prefrontal cortex, and anterior cingulate cortex for each time point separately. Also, we aimed to assess the changes of grey matter of these regions in relationship with changes of BDNF-levels. Hence, we subtracted values of TP1 from TP2, TP1 from TP3, and TP2 from TP3 and correlated the differences in sBDNF levels with the differences in cortical thickness of the prefrontal cortex and hippocampus and amygdala volumes. Correlational analyses were performed between sBDNF levels and BDI scores as well as between sBDNF levels and PSQ20 subscales for the whole group and groups separately. Since psychometric measures were non-normally distributed, additional non-parametric tests (Man-Whitney-U Test) were performed. The significance level was set at *p* < 0.05, statistical values are stated uncorrected. A Bonferroni correction with regards to multiple comparisons was applied separately for the linear mixed models testing imaging data (and here separately for a priori regions and the exploratory analysis), sBDNF concentrations, and psychometric scores. SPSS (IBM Corp. Released 2016. IBM SPSS Statistics for Windows, Version 24.0. Armonk, NY: IBM Corp.) and the jamovi project (2022; Version 2.3; Retrieved from https://www.jamovi.org) was used for statistical analyses.

## Results

The final sample comprised 28 healthy individuals and 18 patients with recurrent MDD (see Tables 1 and 2).

**Table 2:** Demographic information of the study participants.

Group	MDD	HI
N	18	28
Female sex, n (%)	11 (61)	15 (53)
Age, M ± SD	37 ± 10.03	27.96 ± 5.12

*MDD* major depressive disorder, *HI* healthy individuals, *M* mean, *SD* standard deviation. Chi-square test (sex) and *t*-test (age) were performed to test for potential group differences in sex (*p* > 0.1) and age (*p* < 0.01).

### Cortical thickness, surface area, and subcortical volumes across 3 MRI measurements and multiple lockdowns

Due to extensive over- and/or underestimation of volumetric parameters during the FreeSurfer analysis, two measurements (the second measurement of one person with a recurrent MDD and the third measurement of another subject with a recurrent MDD) had to be excluded from the final analyses. According to our hypothesis, we first used cortical thickness and surface area of the anterior cingulate cortex (i.e., caudal anterior cingulate, rostral anterior cingulate), and prefrontal cortex (i.e., superior frontal, rostral middle frontal, caudal middle frontal, pars opercularis, pars triangularis, pars orbitalis, lateral orbitofrontal, medial orbitofrontal, frontal pole) and the volume of the hippocampus and amygdala in a linear mixed model analysis (a normal distribution of residuals of structural MRI data is given). For cortical thickness we found no significant main effect of group (F = 0.19, *p* > 0.1), no significant main effect of time (F = 0.29, *p* > 0.1), and no significant interaction effect of time and participant group (F = 0.28, *p* > 0.1). Similar results were depicted for surface area of these brain areas and the hippocampus and amygdala: no significant main effect of group (F = 0.78, *p* > 0.1) and time (F = 0.69, *p* > 0.1) and no significant interaction effect of time x group (F = 0.05, *p* > 0.1).

In a second step and on an exploratory basis, we performed linear mixed models for cortical thickness and for surface area separately and included data of all 34 ROIs. No main effect of time (cortical thickness: F = 0.16, *p* > 0.1, surface area: F = 0.69, *p* > 0.1) and group (F = 0.03, *p* > 0.1; F1.28, *p* > 0.1) and no interaction effect of time x group (F = 0.43, *p* > 0.1; F = 0.39, *p* > 0.1) were observed. In a linear mixed model including subcortical volumes (thalamus, caudate, putamen, pallidum, hippocampus, amygdala, and accumbens area) and the cerebellar cortex, we likewise found no changes over time (F = 0.06, *p* > 0.1) and group (F = 2.59, *p* > 0.1) and no interaction effects of time and group (F = 0.27, *p* > 0.1). In addition, we performed statistical models for ROIs separately (i.e., for all cortical - cortical thickness and surface area - and subcortical brain regions) and found confirming results: no main effects of time and group and no interaction effect of time x group (all *p* > 0.1).

### BDI and PSQ-20 over the course of multiple lockdowns

**Fig. 2:**
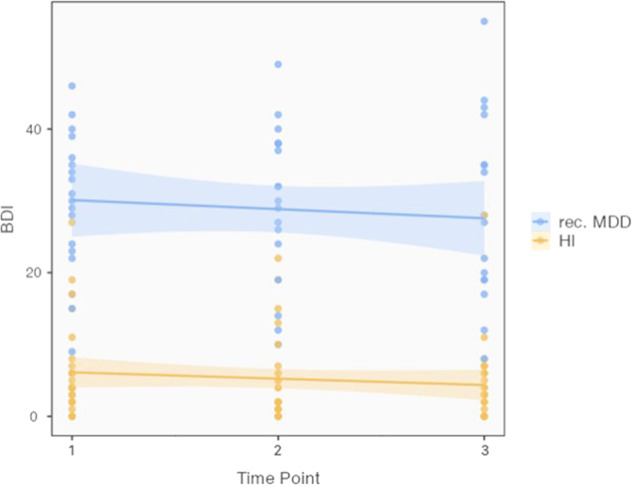
Beck Depression Inventory (BDI-II) scores from 18 patients with recurrent major depressive disorder (rec. MDD) and 28 healthy individuals (HI) across the study phase and 3 study visits. Box-plot (y-axis) demonstrates the distribution of scores. We found significant differences between groups (F = 30.89, *p* < 0.001), but no main effect of time and no interaction effect of time x group (F = 1.36, *p* > 0.1; F = 0.16, *p* > 0.1).

**Fig. 3:**
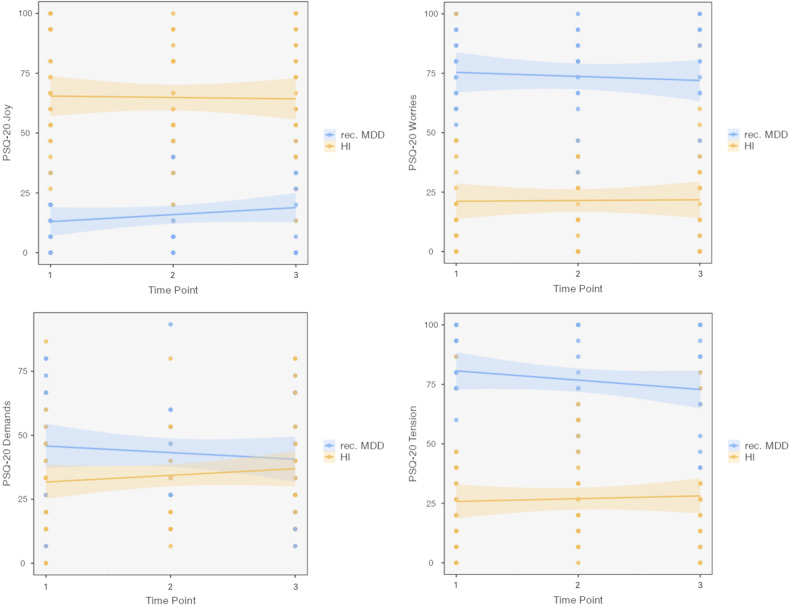
PSQ-20 scores of the subscales worries, tension, joy, and demands from 18 patients with recurrent major depressive disorder (rec. MDD) and 28 healthy individuals (HI). Box-plot (y-axis) demonstrates the distribution of scores. Analysis showed a significant main effect of group for the subscales worries (F = 19.19, *p* < 0.001), tension (F = 34.44, *p* < 0.001), and joy (F = 12.05, *p* = 0.001), but no main effect of group for the PSQ-20 subscale demands (F = 2.13, *p* = 0.15). We found no main effect of time (all *p* > 0.1) and no interaction of time and group (all *p* > 0.1).

We performed linear mixed models for BDI and PSQ-20. Throughout the whole observational period we found a significant main effect of group for BDI (F = 30.89, *p* < 0.001), but no changes over time (F = 1.36, *p* > 0.1) and no interaction effects between time and group (F = 0.16, *p* > 0.1; see Table 3 and Fig. 2). Then, we performed separate linear mixed models for each of the four subscales of the PSQ-20, Worries, Tension, Joy and Demands. For each PSQ-20 subscale model, we observed no main effect of time (Worries: F = 0.19, Tension: F = 0.38, Joy: F = 0.21, Demands: F = 0.02; all *p* > 0.1) and no interaction of time and group (Worries: F = 0.23, Tension: F = 1.76, Joy: F = 1.02, Demands: F = 1.6, all *p* > 0.1). We depicted a main effect of group for three PSQ-20 subscales (Worries: F = 19.19, *p* < 0.001, Tension: F = 34.44, *p* < 0.001, Joy: F = 12.05, *p* = 0.001), but no main effect of group for the PSQ-20 subscale Demands (F = 2.13, *p* = 0.15; also, no significant differences between groups for each of the 3 time points). See Fig. 3 for illustrations of the course of PSQ-20 subscores. Non-parametric tests were performed that depicted similar results as observed with parametric tests (see Supplementary information).

**Table 3:** BDI-II and PSQ-20 subscale scores of patients with an MDD and HI across the three measurement timepoints.

		MDD			HI		
TP	Scale	n	Mean	SD	n	Mean	SD
1	**BDI** ^**a**^	18	29.61	9.86	27	6.07	6.51
	**PSQ20 Worries** ^**b**^	18	75.56	16.01	27	21.98	22.86
	**PSQ20 Tension** ^**b**^	18	81.48	15.60	27	25.19	21.90
	**PSQ20 Joy** ^**b**^	18	12.59	10.45	27	65.93	24.76
	**PSQ20 Demands** ^**b**^	18	45.56	22.67	27	32.10	20.09
2	**BDI** ^**a**^	17	29.35	11.03	27	5.15	5.88
	**PSQ20 Worries** ^**b**^	17	71.76	17.72	27	19.01	18.92
	**PSQ20 Tension** ^**b**^	17	75.29	18.52	27	27.16	18.76
	**PSQ20 Joy** ^**b**^	17	16.86	15.66	27	64.94	22.92
	**PSQ20 Demands** ^**b**^	17	44.71	17.28	27	33.09	15.96
3	**BDI** ^**a**^	16	28.25	14.05	25	4.48	5.72
	**PSQ20 Worries** ^**b**^	16	75.00	21.84	24	23.61	22.41
	**PSQ20 Tension** ^**b**^	16	75.83	18.99	24	28.61	20.66
	**PSQ20 Joy** ^**b**^	16	17.08	15.00	24	63.61	24.94
	**PSQ20 Demands** ^**b**^	16	41.25	20.97	24	38.06	21.13

*MDD* major depressive disorder, *HI* healthy individuals, *TP* time point, *SD* standard deviation, *BDI-II* Beck Depression Inventory II, *PSQ-20* Perceived stress questionnaire.

^a^ BDI-II: Range 0-63; < 13: no depressive symptoms or remitted, 13–19: mild depression, 20–28: moderate depression, ≥ 29: severe depression.

^b^ PSQ-20: Range 0–100 for all subscales; reference values from a sample of psychosomatic patients [20]: PSQ-20 total score: M = 0.52, SD = 0.18; PSQ-20 Worries: M = 0.53, SD = 0.26; PSQ-20 Tension: M = 0.48, SD = 0.12; PSQ-20 Joy: M = 0.37, SD = 0.23; PSQ-20 Demands: M = 0.44, SD = 0.16.

### Serum BDNF over the course of multiple lockdowns

Linear mixed model analysis revealed no main effect of time (F = 0.732, *p* > 0.05) and no interaction effect of time and group (F = 0.633, *p* > 0.05). For the TRD group, mean sBDNF concentration was 11.16 ng/ml (SD ± 3.3) at the 1^st^ visit, 13.28 ng/ml (SD ± 3.1) at the 2^nd^ visit, and 14.62 ng/ml (SD ± 6.4) at the 3^rd^ visit. The values were 11.57 ng/ml (SD ± 3.3), 11.54 ng/ml (SD ± 2.6), and 11.94 ng/ml (SD ± 3.3) for HI, respectively. Further, we found no significant correlations between sBDNF levels and the hippocampus and amygdala, and cortical thickness of prefrontal cortex (all *p* > 0.05). Also, when correlating differences in BDNF- and grey matter values between time points (i.e., TP2-TP1, TP3-TP1, and TP3-TP2), we observed no significant associations (all *p* > 0.05). Also, we correlated sBDNF levels and BDI scores as well as sBDNF and PSQ-20 sub-scale scores for each time point separately. Analysis revealed no significant correlations (r-range from -0.26 to 0.27; all *p* > 0.1). Results did not change when correlations were performed for time points and groups separately (rMDD: r-range -0.35 to 0.16; all *p* > 0.1; HI: r-range from -0.38 to 0.22; all *p* > 0.1).

## Discussion

Within this prospective, quasi-experimental study, we found no significant changes in brain structural, neuroplastic, and psychometric parameters of HIs and patients with a recurrent MDD over time and in response to COVID-19-associated restriction measures in Austria.

Longitudinal studies that focus on pre-, peri-, and post-trauma measurements allow for the investigation of the complete sequence of adaptational processes in response to traumatic and stressful life events. Both stressful and traumatic events have been investigated using neuroimaging techniques to help detect vulnerable groups [17]. In conjunction with other aspects of adaptational processes, neuroimaging findings may thus contribute to discriminate potential biomarkers of a differential susceptibility [18].

Our study period encompassed varying degrees of government response stringency indices [19] and an alternating proximity of measurement time points to the lockdown and opening phases. Despite the relatively high stringency index in Austria [19], we did not find any indication of heightened stress or depressive symptoms in the HIs on a group level, but clinically relevant scores in the BDI-II and PSQ-20 subscales worries, tension, and joys in patients with a recurrent MDD (indication for clinically relevant PSQ-20 score with regard to a sample of patients with a psychosomatic illness) [20]. Significant cross-sectional differences between the two groups were found for BDI-II as well as for the PSQ-20 subscales worries, tension and joy, but not for the PSQ-20 subscale external demands (see Table 2). Also, we did not observe any statistically significant longitudinal changes in psychometric scores across the three measurement time points. Meta-analytical data from the initial phase of the pandemic suggest an increase of mental health symptoms during the first lockdown in March and April 2020 and a consecutive decline to pre-pandemic levels in that summer [21]. The relatively high level of demands experienced by HIs might be associated with increasing external stressors in the HIs during the pandemic, for example, due to home office and childcare. In MDDs, in contrast, a decrease of demands during the pandemic could be hypothesised, e.g., due to a reduction of social stressors. Adaptational effects led to decreased distress after the first lockdown in both healthy individuals and persons with a known psychiatric disorder [22]. Our patient population comprised individuals with a recurrent MDD, with clinically relevant scores of stress and depression that did, however, not change in terms of severity throughout the observational period. Along these lines, a study on psychiatric outpatients in Germany finds unchanged psychiatric symptoms and a reduction in psychosocial burden in the course of the pandemic [23], suggesting potential resilience factors that influenced this development. Cohrdes et al. defined at least four groups with distinct patterns during the COVID-19 pandemic, implicating that people with a specific set of factors such as good physical health, openness and perception of social support had a higher probability of a resilient trajectory [24]. Also, a study on mental health status in a large German sample described vulnerability to lockdowns only in a subsample of previously mentally healthy individuals, without a general negative effect on mental health [25]. Data by van de Weijer et al. suggest that restriction measures for the whole population increased the effect of non-shared environmental and individual factors on quality of life [26]. While a certain degree of heterogeneity and variability of lifestyles during the COVID-19 pandemic and lockdowns might have influenced our results, both negative *and* positive lifestyle changes can have an effect on psychological distress during the pandemic and restriction measures [27]. Another contributor to heightened stress levels might have been limited medical care and availability of specialised mental health services during restriction measures, such as maintenance electroconvulsive therapy [28], with discontinuations of psychotherapy and consultations during lockdown periods also in Austria [29]. However, restriction measures seemed particularly relevant for treatment initiation for patients with first-episode symptoms, while patients with prior treatment were less affected [30]. Also, mental health services adapted fast in the light of COVID-19, as seen in the surge of helplines and telehealth services [31].

Alongside the psychometric scores, we observed no significant changes across the observational period in sBNDF levels of both patients and HIs. Social isolation as well as different forms of stress can influence BDNF levels [32]. Also, depressive episodes may contribute to an attenuation of BDNF levels [33]. Considering the clinically relevant stress and depressive symptom scores in patients with MDD, the lack of any group differences in sBDNF levels seems surprising. One explanation might be the chronic administration of antidepressant medication that has been shown to increase sBDNF levels of healthy individuals [33]. Moreover, sBDNF levels in HIs might have dropped as a results of the first lockdown in March 2020 and were slow to recover, as reported by Stahn and colleagues, where the effects of social isolation in the course of a 14-month Antarctic exploration were investigated [11].

Neuroimaging studies show relatively robust cross-sectional findings when comparing patients with MDD to HIs, with small but significant effect sizes [34, 35]. However, a recent comparison of HIs and patients with MDD showed no longitudinal structural differences [36]. In contrast, previous imaging studies with populations ranging from 10 to 340 subjects find structural differences and changes associated with single or repeated traumatic and stressful experiences [17]. These investigations highlight the effects of traumatic or stressful life events on the grey matter of the limbic system and connected regions, and thus also underscore the measurability of these changes with structural MRI. Currently, there is a void of studies focusing on the effects of COVID-19-associated restriction measures on brain structure in patients and healthy individuals. In a rare investigation on volumetric changes associated with mitigation strategies during the COVID-19 pandemic, Salomon et al. found increases in the volumes of both amygdalae, putamen and ventral anterior temporal cortices in healthy subjects compared to before the pandemic [37], whereas the changes in the amygdalae diminished with time following lockdown. In our sample of patients and HIs, we did not observe any adaptational processes, such as changes in amygdala volume in HIs, after the first or second measurements as described previously [37]. Studies on recently traumatised persons (i.e., less than a year) do not necessarily find significant volumetric changes in the hippocampus or the amygdala [38]. For example, van Wingen et al. found no significant volumetric changes over time between soldiers deployed to Afghanistan and during war for four months and control subjects, in a study with a comparable sample size [39]. In our study, the perceived stress levels in patients with a recurrent MDD appear to reflect clinically relevant stress levels throughout the observational period assessed with PSQ-20 and its subscales worries, tension and joy in comparison to patients with a psychosomatic illness [20]. However, we did not observe significant differences of stress levels over the course of multiple COVID-19-associated lockdowns in all study subjects. The finding of no significant changes of psychometric scores might indicate that the stress through lockdowns might not have been particularly severe and that a traumatic threshold was not surpassed throughout the pandemic, thus potentially not entailing comparable volumetric changes as seen after single traumatic life events [10]. In addition, previous treatment regimens might have attenuated the effects, since antidepressive medication is found to reverse neuroplastic and immunologic influences on neurobiology that is important for the evolvement of mental disorders [8]. Some patients had previously received ECT, which might have already altered cortical thickness [40]. A more structured routine with a decrease in external stressors during restriction measure periods, the introduction of additional mental health services, and a consecutive decrease of compliance to restriction measures might have contributed to a reduction of the traumatic threshold. Of note, a comparison of Austria and the UK during the pandemic hinted towards an association of COVID-19 incidence and mortality with the occurrence of psychological symptoms [41]. The Austrian healthcare system was confronted with comparably moderate incidence and mortality rates especially at the beginning of the pandemic. This might have reduced the impact of restriction measures on neuroplasticity and brain structure, and also limit the translatability of our data to other geographic regions.

However, our findings of elevated stress in individuals with MDD over the whole observational period and no significant changes in depressive symptoms in both groups are in line with recent meta-analytical evidence on the progression of mental health symptoms during the COVID-19 pandemic [42, 43].

### Limitations

The current study followed a naturalistic, observational design, with the first measurement performed already about five months after the first COVID-19-associated lockdown. As such, there was no intervention per se and no control group that had not been subjected to mitigation strategies. However, we implemented a quasi-experimental design and were thereby able to compare mentally healthy persons to individuals with a recurrent MDD. It should be noted that the results of our analyses relate to a specific subgroup and might only partly be translated to e.g., therapy-naïve patients having their first depressive episode. Pervading our results are non-significant changes of psychometric, neuroplastic and brain structural markers across ten months of the COVID-19 pandemic in Austria and multiple lockdowns with a varying degree of stringency. With the first examination five months after the first lockdown we might have missed the effects of early adaptational processes, including immunologic, neuroplastic and neuroendocrine mechanisms that can impact brain structure [8]. In recording peri-pandemic data an interpretation of the results as resilience is difficult. However, the concept of resilience comprises long-lasting adaptational processes that ultimately allow for the restitution to or maintenance of a state of mental well-being [17]. Since the COVID-19 pandemic constitutes a new and constantly developing healthcare issue and a potentially new trauma type [3], it appears complex to assess whether our measurements were already performed post- or still peri-traumatically. Our findings might thus reflect an already adapted state or an ongoing adaption of neurobiology, which requires further longitudinal observations. Although potentially relevant for our findings and the assessment of the effects of lifestyle changes through restriction measures, we could not include data on the compliance with restriction measures and data on the digital phenotype such as walking distance and screen time [44, 45]. Also, despite our naturalistic quasi-experimental setting, inclusion into this study might have biased psychometric outcome variables, since the study design included regular visits and ongoing, assured care with the possibility of receiving specialised treatment not available elsewhere such as the adaptation and optimisation of treatment regimens (e.g., treatment with stimulants or esketamine). While comparable to other neuroimaging studies investigating the effects of traumatic and stressful life events [9–11, 17, 46, 47], our study population might have been too small to assess changes in brain structure and neuroplasticity during the COVID-19 pandemic.

## Conclusion

This is the first study investigating potential neurobiological effects of multiple COVID-19 pandemic lockdowns on different aspects of adaptational processes in patients with a recurrent MDD and healthy individuals in Austria. While we are aware of the sample size as a potential limitation, we did not find any significant differences from after the 1^st^ lockdown to after the 2^nd^ and multiple lockdowns in brain structural, neuroplastic and psychometric parameters of patients and healthy individuals, despite the extensive restriction measures not only for infected people but for the general population and the non-negligible, far-reaching global implications of the COVID-19 pandemic. The lack of any changes across lockdowns, and especially the lack of an exacerbation of psychiatric symptoms, might point towards adaptational processes and resilience. Our data are in line with recent meta-analytical evidence and inform future meta-analyses about longitudinal effects of restriction measures on psychiatric symptoms.

### Supplementary information

Supplementary information

## Data Availability

The data that support the findings of this study are available on request from the corresponding author, TV. The data are not publicly available due to data protection laws.
