# Supplementary material for: Effects of lockdowns on neurobiological and psychometric parameters in unipolar depression during the COVID-19 pandemic
Source: Transl Psychiatry. 2024 Jan 19;14:42. doi: 10.1038/s41398-024-02733-1 (PMC10798945; doi:10.1038/s41398-024-02733-1)
Supplement: Supplementary file 1 — Supplementary information [file 41398_2024_2733_MOESM1_ESM.pdf]

Results

Descriptives - Psychometric Measures and Serum BDNF Concentrations

Descriptives

	Participant Group	Time Point	N	Missing	Mean	SD	Minimum	Maximum
BDI	rec MDD	1	18	0	29.61	9.86	9	46
		2	18	0	29.83	10.89	10	49
		3	17	1	27.06	14.47	0	55
	Healthy Ind.	1	27	1	6.07	6.51	0	27
		2	26	2	5.35	5.91	0	22
		3	26	2	4.31	5.67	0	28
PSQ-20 Worries	rec MDD	1	18	0	75.56	16.01	46.67	100.0
		2	18	0	73.33	18.44	33.33	100.0
		3	17	1	72.16	24.18	26.67	100.0
	Healthy Ind.	1	27	1	21.98	22.86	0.00	100.0
		2	26	2	19.74	18.90	0.00	80.0
		3	25	3	22.67	22.44	0.00	86.7
PSQ-20 Tension	rec MDD	1	18	0	81.48	15.60	40.00	100.0
		2	18	0	75.19	17.98	46.67	100.0
		3	17	1	73.73	20.34	40.00	100.0
	Healthy Ind.	1	27	1	25.19	21.90	0.00	86.7
		2	26	2	28.21	18.31	0.00	66.7
		3	25	3	27.47	21.02	0.00	80.0
PSQ-20 Joy	rec MDD	1	18	0	12.59	10.45	0.00	33.3
		2	18	0	16.67	15.21	0.00	46.7
		3	17	1	18.43	15.55	0.00	46.7
	Healthy Ind.	1	27	1	65.93	24.76	0.00	100.0
		2	26	2	63.85	22.64	13.33	100.0
		3	25	3	64.80	25.13	13.33	100.0
PSQ-20 Demands	rec MDD	1	18	0	45.56	22.67	6.67	80.0
		2	18	0	43.70	17.30	26.67	93.3
		3	17	1	40.39	20.61	6.67	73.3
	Healthy Ind.	1	27	1	32.10	20.09	0.00	86.7
		2	26	2	33.59	16.05	6.67	80.0
		3	25	3	37.33	20.99	6.67	80.0
BDNF	rec MDD	1	18	0	11.16	3.32	6.79	18.2
		2	17	1	13.28	3.09	9.27	18.9
		3	15	3	14.62	6.40	5.47	30.7
	Healthy Ind.	1	27	1	11.69	2.14	7.33	15.9
		2	26	2	11.54	2.59	7.65	18.1
		3	24	4	11.94	3.34	7.59	24.2

BDI and PSQ-20 over the course of multiple lockdowns

We applied the Mann-Whitney-U-Test to assess differences in BDI and PSQ-20 (Subscales) within groups (i.e. TP2-1, TP3-1, TP3-2) as well as between groups (MDD vs. HI) at each time point.

Analysis revealed no changes in BDI and PSQ-20 for patients with MDD and HI separately across time (all $p>0.1$). Except for the PSQ-20 subscale demands (TP1 to 3, $p>0.1$), we found significant differences at each time point between MDD vs. HI (all $p<0.001$).

Descriptives - Surface Area of Cortical Brain Regions

	Participant Group	Time Point	N	Missing	Mean	SD	Minimum	Maximum
BANKSSTS AREA	rec MDD	1	18	0	689	105.7	581	966
		2	17	1	692	104.9	583	959
		3	16	2	693	109.9	579	966
	Healthy Ind.	1	28	0	685	75.5	547	843
		2	26	2	684	80.5	546	855
		3	26	2	693	76.7	543	844
CAUDALANTERIORCINGULATE AREA	rec MDD	1	18	0	498	75.4	357	646
		2	17	1	496	76.1	358	637
		3	16	2	497	80.1	356	632
	Healthy Ind.	1	28	0	473	74.2	337	625
		2	26	2	478	75.2	339	625
		3	26	2	472	77.9	335	629
CAUDALMIDDLEFRONTAL AREA	rec MDD	1	18	0	1522	113.8	1301	1680
		2	17	1	1533	102.4	1356	1679
		3	16	2	1496	107.7	1303	1679
	Healthy Ind.	1	28	0	1525	203.8	1129	2010
		2	26	2	1520	209.6	1109	2013
		3	26	2	1538	184.5	1220	1982
CUNEUS AREA	rec MDD	1	18	0	1056	104.4	922	1276
		2	17	1	1037	92.4	921	1221
		3	16	2	1056	112.2	912	1299
	Healthy Ind.	1	28	0	1101	152.8	790	1391
		2	26	2	1111	155.6	800	1407
		3	26	2	1086	152.7	774	1399
ENTORHINAL AREA	rec MDD	1	18	0	317	42.1	213	385
		2	17	1	316	44.4	216	378
		3	16	2	310	42.2	218	378
	Healthy Ind.	1	28	0	297	56.6	221	425
		2	26	2	289	53.8	210	429
		3	26	2	299	58.5	225	427
FUSIFORM AREA	rec MDD	1	18	0	2127	138.3	1867	2341
		2	17	1	2121	143.3	1868	2332
		3	16	2	2123	142.2	1871	2331
	Healthy Ind.	1	28	0	2131	160.5	1817	2514
		2	26	2	2133	162.0	1797	2520
		3	26	2	2138	160.3	1807	2495
INFERIORPARIETAL AREA	rec MDD	1	18	0	3567	384.7	2921	4476
		2	17	1	3582	341.6	2995	4159
		3	16	2	3566	353.7	2977	4268
	Healthy Ind.	1	28	0	3475	348.2	2936	4264
		2	26	2	3466	367.3	2911	4299
		3	26	2	3488	375.9	2946	4246
INFERIORTEMPORAL AREA	rec MDD	1	18	0	2397	275.8	1915	3045
		2	17	1	2417	269.1	1914	3035
		3	16	2	2402	274.1	1947	3072
	Healthy Ind.	1	28	0	2333	208.5	1975	2764
		2	26	2	2344	218.1	1963	2786
		3	26	2	2360	199.3	2042	2816
ISTHMUSCINGULATE AREA	rec MDD	1	18	0	630	82.2	500	824
		2	17	1	636	80.6	505	821

Descriptives

	Participant Group	Time Point	N	Missing	Mean	SD	Minimum	Maximum
		3	16	2	629	74.0	523	810
	Healthy Ind.	1	28	0	661	73.1	495	791
		2	26	2	665	79.0	491	788
		3	26	2	659	71.1	503	783
LATERALOCCIPITAL AREA	rec MDD	1	18	0	3608	322.0	3063	4171
		2	17	1	3593	326.8	3075	4174
		3	16	2	3596	285.6	3122	4124
	Healthy Ind.	1	28	0	3775	370.7	3083	4469
		2	26	2	3782	363.7	3161	4464
		3	26	2	3753	349.0	3107	4409
LATERALORBITOFRONTAL AREA	rec MDD	1	18	0	1850	167.7	1536	2202
		2	17	1	1840	170.5	1482	2128
		3	16	2	1851	147.0	1676	2193
	Healthy Ind.	1	28	0	1884	137.5	1647	2165
		2	26	2	1885	140.3	1646	2121
		3	26	2	1879	139.3	1650	2126
LINGUAL AREA	rec MDD	1	18	0	2120	278.5	1566	2601
		2	17	1	2072	262.4	1525	2446
		3	16	2	2107	285.3	1522	2608
	Healthy Ind.	1	28	0	2173	258.5	1628	2619
		2	26	2	2178	260.4	1604	2627
		3	26	2	2163	259.4	1627	2697
MEDIALORBITOFRONTAL AREA	rec MDD	1	18	0	1406	118.4	1233	1618
		2	17	1	1399	118.5	1245	1618
		3	16	2	1395	109.8	1234	1585
	Healthy Ind.	1	28	0	1418	101.0	1249	1637
		2	26	2	1429	103.4	1237	1630
		3	26	2	1421	111.4	1127	1626
MIDDLETEMPORAL AREA	rec MDD	1	18	0	2404	229.8	1980	2845
		2	17	1	2413	230.8	1964	2817
		3	16	2	2407	208.8	2065	2820
	Healthy Ind.	1	28	0	2380	170.4	2099	2953
		2	26	2	2377	172.0	2135	2914
		3	26	2	2382	167.6	2116	2948
PARAHIPPOCAMPAL AREA	rec MDD	1	18	0	432	38.4	374	514
		2	17	1	435	40.2	368	520
		3	16	2	433	40.9	380	513
	Healthy Ind.	1	28	0	455	51.1	390	576
		2	26	2	452	48.0	387	585
		3	26	2	456	50.3	389	580
PARACENTRAL AREA	rec MDD	1	18	0	986	77.6	827	1137
		2	17	1	985	81.0	828	1140
		3	16	2	980	78.2	822	1127
	Healthy Ind.	1	28	0	956	77.6	783	1110
		2	26	2	951	75.9	787	1106
		3	26	2	952	80.7	759	1116
PARSOPERCULARIS AREA	rec MDD	1	18	0	1063	129.7	877	1400
		2	17	1	1070	120.4	874	1383
		3	16	2	1060	130.4	874	1393
	Healthy Ind.	1	28	0	1054	144.7	815	1495

Descriptives

	Participant Group	Time Point	N	Missing	Mean	SD	Minimum	Maximum
		2	26	2	1059	137.4	824	1486
		3	26	2	1046	143.0	814	1479
PARSORBITALIS AREA	rec MDD	1	18	0	550	77.4	395	671
		2	17	1	548	79.2	393	670
		3	16	2	549	67.0	405	666
	Healthy Ind.	1	28	0	554	48.3	457	646
		2	26	2	557	50.5	455	645
		3	26	2	544	45.4	455	624
PARSTRIANGULARIS AREA	rec MDD	1	18	0	1037	107.9	816	1318
		2	17	1	1027	106.8	812	1321
		3	16	2	1045	101.0	932	1306
	Healthy Ind.	1	28	0	1026	121.5	819	1318
		2	26	2	1030	118.4	829	1285
		3	26	2	1010	120.5	812	1299
PERICALCARINE AREA	rec MDD	1	18	0	1016	154.8	768	1273
		2	17	1	989	146.3	763	1263
		3	16	2	1007	158.1	762	1272
	Healthy Ind.	1	28	0	1056	191.8	724	1606
		2	26	2	1057	197.6	711	1588
		3	26	2	1029	164.0	700	1412
POSTCENTRAL AREA	rec MDD	1	18	0	2711	356.0	2144	3320
		2	17	1	2750	345.2	2164	3360
		3	16	2	2700	311.9	2207	3194
	Healthy Ind.	1	28	0	2739	241.5	2360	3198
		2	26	2	2704	223.4	2381	3123
		3	26	2	2723	242.0	2341	3141
POSTERIORCINGULATE AREA	rec MDD	1	18	0	843	83.7	686	1013
		2	17	1	848	81.6	705	1009
		3	16	2	845	84.3	688	1006
	Healthy Ind.	1	28	0	805	64.7	691	982
		2	26	2	808	67.9	693	991
		3	26	2	806	63.2	695	977
PRECENTRAL AREA	rec MDD	1	18	0	3270	267.8	2846	3869
		2	17	1	3285	250.9	2958	3769
		3	16	2	3250	248.7	2890	3773
	Healthy Ind.	1	28	0	3281	262.5	2893	3860
		2	26	2	3276	265.4	2905	3850
		3	26	2	3270	263.3	2894	3851
PRECUNEUS AREA	rec MDD	1	18	0	2597	211.5	2288	3104
		2	17	1	2609	208.8	2283	3102
		3	16	2	2589	192.4	2362	3077
	Healthy Ind.	1	28	0	2652	264.1	2260	3238
		2	26	2	2654	271.8	2251	3267
		3	26	2	2640	275.7	2223	3259
ROSTRALANTERIORCINGULATE AREA	rec MDD	1	18	0	510	55.8	417	626
		2	17	1	509	55.4	416	610
		3	16	2	504	48.6	441	595
	Healthy Ind.	1	28	0	495	47.4	421	661
		2	26	2	495	45.4	409	649
		3	26	2	492	44.7	427	644

Descriptives

	Participant Group	Time Point	N	Missing	Mean	SD	Minimum	Maximum
ROSTRALMIDDLEFRONTAL AREA	rec MDD	1	18	0	4168	438.1	3399	4906
		2	17	1	4108	410.6	3353	4886
		3	16	2	4111	393.2	3358	4841
	Healthy Ind.	1	28	0	4207	399.1	3314	5042
		2	26	2	4190	392.5	3290	4986
		3	26	2	4197	387.0	3331	4952
SUPERIORFRONTAL AREA	rec MDD	1	18	0	4989	372.5	4379	5817
		2	17	1	4995	366.2	4476	5774
		3	16	2	4917	346.0	4393	5785
	Healthy Ind.	1	28	0	5006	389.4	4287	5930
		2	26	2	5007	390.9	4286	5883
		3	26	2	4958	391.6	4294	5841
SUPERIORPARIETAL AREA	rec MDD	1	18	0	3722	465.1	3238	5183
		2	17	1	3738	406.4	3274	4811
		3	16	2	3732	450.7	3208	5078
	Healthy Ind.	1	28	0	3714	435.7	2970	4694
		2	26	2	3691	457.6	2757	4745
		3	26	2	3695	468.4	2877	4710
SUPERIORTEMPORAL AREA	rec MDD	1	18	0	2656	200.2	2276	3122
		2	17	1	2661	205.5	2261	3129
		3	16	2	2664	186.9	2387	3096
	Healthy Ind.	1	28	0	2694	192.5	2348	3139
		2	26	2	2682	174.0	2352	3014
		3	26	2	2678	195.5	2357	3125
SUPRAMARGINAL AREA	rec MDD	1	18	0	2600	335.6	2076	3227
		2	17	1	2634	306.9	2265	3216
		3	16	2	2576	309.5	2074	3172
	Healthy Ind.	1	28	0	2716	324.1	2032	3431
		2	26	2	2701	321.6	2029	3455
		3	26	2	2709	337.4	2020	3423
FRONTALPOLE AREA	rec MDD	1	18	0	217	27.1	173	263
		2	17	1	215	27.9	179	269
		3	16	2	212	22.7	182	263
	Healthy Ind.	1	28	0	225	19.0	198	272
		2	26	2	226	18.7	201	271
		3	26	2	221	18.3	193	266
TEMPORALPOLE AREA	rec MDD	1	18	0	348	35.1	284	410
		2	17	1	336	30.1	285	396
		3	16	2	343	33.6	290	406
	Healthy Ind.	1	28	0	332	30.2	279	397
		2	26	2	332	30.1	290	398
		3	26	2	335	29.9	285	393
TRANSVERSETEMPORAL AREA	rec MDD	1	18	0	275	30.7	225	332
		2	17	1	273	29.7	219	324
		3	16	2	274	31.9	213	329
	Healthy Ind.	1	28	0	277	29.2	221	346
		2	26	2	276	28.3	229	338
		3	26	2	275	28.6	226	331
INSULA AREA	rec MDD	1	18	0	1522	120.1	1335	1746
		2	17	1	1517	116.0	1332	1730

Descriptives

	Participant Group	Time Point	N	Missing	Mean	SD	Minimum	Maximum
		3	16	2	1525	120.0	1342	1721
	Healthy Ind.	1	28	0	1587	143.6	1337	1827
		2	26	2	1588	147.1	1326	1836
		3	26	2	1578	146.2	1301	1839

Descriptives - Cortical Tickness of Cortical Brain Regions

	Participant Group	Time Point	N	Missing	Mean	SD	Minimum	Maximum
BANKSSTS THICKNESS	rec MDD	1	18	0	2.56	0.1469	2.31	2.80
		2	17	1	2.55	0.1556	2.31	2.78
		3	16	2	2.56	0.1570	2.34	2.80
	Healthy Ind.	1	28	0	2.63	0.1182	2.40	2.83
		2	26	2	2.63	0.1091	2.42	2.82
		3	26	2	2.64	0.1118	2.44	2.88
CAUDALANTERIORCINGULATE THICKNESS	rec MDD	1	18	0	2.47	0.1364	2.21	2.72
		2	17	1	2.45	0.1332	2.23	2.71
		3	16	2	2.48	0.1424	2.20	2.73
	Healthy Ind.	1	28	0	2.52	0.1275	2.27	2.77
		2	26	2	2.53	0.1283	2.31	2.77
		3	26	2	2.51	0.1231	2.31	2.73
CAUDALMIDDLEFRONTAL THICKNESS	rec MDD	1	18	0	2.63	0.1427	2.39	2.90
		2	17	1	2.61	0.1247	2.39	2.79
		3	16	2	2.65	0.1420	2.42	2.89
	Healthy Ind.	1	28	0	2.74	0.0917	2.57	2.96
		2	26	2	2.74	0.0796	2.58	2.92
		3	26	2	2.75	0.0792	2.57	2.91
CUNEUS THICKNESS	rec MDD	1	18	0	1.92	0.1106	1.77	2.16
		2	17	1	1.92	0.1054	1.72	2.08
		3	16	2	1.91	0.1330	1.66	2.16
	Healthy Ind.	1	28	0	1.98	0.1020	1.74	2.15
		2	26	2	1.97	0.1078	1.76	2.18
		3	26	2	1.98	0.1037	1.70	2.13
ENTORHINAL THICKNESS	rec MDD	1	18	0	3.25	0.3073	2.69	3.96
		2	17	1	3.25	0.2794	2.82	3.88
		3	16	2	3.31	0.2623	2.92	3.92
	Healthy Ind.	1	28	0	3.24	0.2017	2.88	3.67
		2	26	2	3.23	0.2079	2.85	3.73
		3	26	2	3.23	0.2347	2.75	3.72
FUSIFORM THICKNESS	rec MDD	1	18	0	2.77	0.1461	2.59	3.06
		2	17	1	2.74	0.1240	2.55	2.98
		3	16	2	2.78	0.1519	2.55	3.06
	Healthy Ind.	1	28	0	2.84	0.1027	2.63	3.02
		2	26	2	2.84	0.0964	2.64	2.99
		3	26	2	2.85	0.0944	2.65	3.00
INFERIORPARIETAL THICKNESS	rec MDD	1	18	0	2.54	0.1233	2.27	2.74
		2	17	1	2.53	0.0970	2.34	2.71
		3	16	2	2.55	0.1132	2.34	2.69
	Healthy Ind.	1	28	0	2.61	0.1011	2.39	2.79
		2	26	2	2.61	0.0885	2.39	2.75
		3	26	2	2.62	0.0865	2.45	2.75
INFERIORTEMPORAL THICKNESS	rec MDD	1	18	0	2.81	0.1165	2.54	3.02

Descriptives

	Participant Group	Time Point	N	Missing	Mean	SD	Minimum	Maximum
		2	17	1	2.79	0.1129	2.53	2.96
		3	16	2	2.81	0.1230	2.60	3.00
	Healthy Ind.	1	28	0	2.86	0.1090	2.55	3.08
		2	26	2	2.86	0.1052	2.55	3.05
		3	26	2	2.87	0.0923	2.73	3.07
ISTHMUSCINGULATE THICKNESS	rec MDD	1	18	0	2.42	0.1840	2.14	2.76
		2	17	1	2.39	0.1769	2.11	2.71
		3	16	2	2.41	0.1830	2.14	2.72
	Healthy Ind.	1	28	0	2.44	0.1647	2.16	2.73
		2	26	2	2.43	0.1741	2.15	2.73
		3	26	2	2.43	0.1657	2.14	2.73
LATERALOCCIPITAL THICKNESS	rec MDD	1	18	0	2.10	0.0855	1.98	2.31
		2	17	1	2.10	0.0621	2.01	2.23
		3	16	2	2.10	0.0862	1.99	2.36
	Healthy Ind.	1	28	0	2.16	0.1082	1.97	2.37
		2	26	2	2.16	0.1040	1.98	2.33
		3	26	2	2.17	0.1007	1.99	2.35
LATERALORBITOFRONTAL THICKNESS	rec MDD	1	18	0	2.73	0.1132	2.44	2.91
		2	17	1	2.72	0.0847	2.59	2.93
		3	16	2	2.75	0.1031	2.56	2.92
	Healthy Ind.	1	28	0	2.80	0.1015	2.65	3.04
		2	26	2	2.81	0.0989	2.66	3.04
		3	26	2	2.83	0.0978	2.64	3.04
LINGUAL THICKNESS	rec MDD	1	18	0	1.99	0.1093	1.73	2.21
		2	17	1	2.00	0.0831	1.81	2.19
		3	16	2	1.99	0.1080	1.73	2.22
	Healthy Ind.	1	28	0	2.07	0.1079	1.83	2.29
		2	26	2	2.06	0.1178	1.81	2.34
		3	26	2	2.06	0.1117	1.84	2.25
MEDIALORBITOFRONTAL THICKNESS	rec MDD	1	18	0	2.54	0.0904	2.36	2.78
		2	17	1	2.53	0.0782	2.36	2.65
		3	16	2	2.57	0.0909	2.46	2.83
	Healthy Ind.	1	28	0	2.64	0.0969	2.44	2.81
		2	26	2	2.64	0.0807	2.47	2.82
		3	26	2	2.66	0.0940	2.54	2.95
MIDDLETEMPORAL THICKNESS	rec MDD	1	18	0	2.89	0.1342	2.60	3.10
		2	17	1	2.88	0.1299	2.59	3.07
		3	16	2	2.90	0.1361	2.61	3.08
	Healthy Ind.	1	28	0	2.98	0.1191	2.77	3.22
		2	26	2	2.98	0.1123	2.77	3.19
		3	26	2	3.00	0.1095	2.78	3.18
PARAHIPPOCAMPAL THICKNESS	rec MDD	1	18	0	2.78	0.1820	2.40	3.08
		2	17	1	2.76	0.1811	2.38	3.09

Descriptives

	Participant Group	Time Point	N	Missing	Mean	SD	Minimum	Maximum
		3	16	2	2.78	0.1753	2.43	3.06
	Healthy Ind.	1	28	0	2.77	0.2100	2.29	3.27
		2	26	2	2.75	0.2307	2.24	3.29
		3	26	2	2.77	0.2295	2.27	3.31
PARACENTRAL THICKNESS	rec MDD	1	18	0	2.51	0.1143	2.30	2.75
		2	17	1	2.50	0.0867	2.40	2.71
		3	16	2	2.53	0.1227	2.31	2.77
	Healthy Ind.	1	28	0	2.63	0.1273	2.42	2.91
		2	26	2	2.63	0.1101	2.40	2.82
		3	26	2	2.63	0.1128	2.46	2.89
PARSOPERCULARIS THICKNESS	rec MDD	1	18	0	2.68	0.1620	2.44	2.95
		2	17	1	2.65	0.1493	2.39	2.93
		3	16	2	2.67	0.1626	2.41	2.92
	Healthy Ind.	1	28	0	2.78	0.1261	2.56	3.13
		2	26	2	2.79	0.1129	2.53	3.02
		3	26	2	2.80	0.1082	2.59	3.03
PARSORBITALIS THICKNESS	rec MDD	1	18	0	2.82	0.1433	2.57	3.07
		2	17	1	2.80	0.1188	2.59	3.05
		3	16	2	2.84	0.1410	2.61	3.09
	Healthy Ind.	1	28	0	2.87	0.1173	2.65	3.18
		2	26	2	2.87	0.1055	2.73	3.14
		3	26	2	2.89	0.1166	2.72	3.15
PARSTRIANGULARIS THICKNESS	rec MDD	1	18	0	2.54	0.1387	2.30	2.75
		2	17	1	2.52	0.1286	2.29	2.75
		3	16	2	2.54	0.1458	2.33	2.76
	Healthy Ind.	1	28	0	2.63	0.1306	2.40	2.87
		2	26	2	2.63	0.1270	2.41	2.89
		3	26	2	2.64	0.1330	2.40	2.90
PERICALCARINE THICKNESS	rec MDD	1	18	0	1.62	0.1591	1.28	2.03
		2	17	1	1.65	0.1566	1.29	1.89
		3	16	2	1.59	0.1735	1.24	1.96
	Healthy Ind.	1	28	0	1.69	0.1534	1.43	2.04
		2	26	2	1.68	0.1479	1.40	1.97
		3	26	2	1.70	0.1403	1.37	1.96
POSTCENTRAL THICKNESS	rec MDD	1	18	0	2.26	0.1562	1.99	2.53
		2	17	1	2.23	0.1427	2.05	2.52
		3	16	2	2.25	0.1676	2.05	2.51
	Healthy Ind.	1	28	0	2.30	0.1059	2.05	2.50
		2	26	2	2.31	0.0949	2.05	2.50
		3	26	2	2.32	0.0778	2.18	2.48
POSTERIORCINGULATE THICKNESS	rec MDD	1	18	0	2.45	0.1151	2.23	2.74
		2	17	1	2.44	0.1078	2.18	2.66
		3	16	2	2.46	0.1311	2.20	2.76

Descriptives

	Participant Group	Time Point	N	Missing	Mean	SD	Minimum	Maximum
	Healthy Ind.	1	28	0	2.51	0.1196	2.28	2.77
		2	26	2	2.51	0.1247	2.26	2.73
		3	26	2	2.50	0.1016	2.35	2.66
PRECENTRAL THICKNESS	rec MDD	1	18	0	2.62	0.1299	2.38	2.89
		2	17	1	2.63	0.1142	2.44	2.84
		3	16	2	2.62	0.1237	2.43	2.89
	Healthy Ind.	1	28	0	2.74	0.1506	2.41	3.01
		2	26	2	2.74	0.1416	2.45	3.01
		3	26	2	2.74	0.1502	2.41	3.02
PRECUNEUS THICKNESS	rec MDD	1	18	0	2.50	0.1234	2.28	2.78
		2	17	1	2.47	0.0916	2.30	2.59
		3	16	2	2.49	0.1258	2.28	2.74
	Healthy Ind.	1	28	0	2.55	0.1070	2.34	2.77
		2	26	2	2.55	0.1023	2.33	2.77
		3	26	2	2.56	0.1059	2.36	2.80
ROSTRALANTERIORCINGULATE THICKNESS	rec MDD	1	18	0	2.79	0.1180	2.61	3.08
		2	17	1	2.78	0.0924	2.57	2.91
		3	16	2	2.81	0.1172	2.60	3.10
	Healthy Ind.	1	28	0	2.89	0.1497	2.59	3.15
		2	26	2	2.88	0.1605	2.56	3.18
		3	26	2	2.89	0.1517	2.57	3.11
ROSTRALMIDDLEFRONTAL THICKNESS	rec MDD	1	18	0	2.45	0.1107	2.29	2.76
		2	17	1	2.43	0.0766	2.27	2.55
		3	16	2	2.47	0.1071	2.35	2.75
	Healthy Ind.	1	28	0	2.53	0.0757	2.40	2.67
		2	26	2	2.54	0.0730	2.41	2.63
		3	26	2	2.54	0.0764	2.38	2.64
SUPERIORFRONTAL THICKNESS	rec MDD	1	18	0	2.78	0.1213	2.54	3.06
		2	17	1	2.76	0.0985	2.55	2.90
		3	16	2	2.79	0.1296	2.56	3.06
	Healthy Ind.	1	28	0	2.88	0.0975	2.66	3.08
		2	26	2	2.88	0.0864	2.67	3.03
		3	26	2	2.88	0.0996	2.68	3.07
SUPERIORPARIETAL THICKNESS	rec MDD	1	18	0	2.31	0.1197	2.04	2.52
		2	17	1	2.29	0.0838	2.14	2.49
		3	16	2	2.31	0.1279	2.12	2.54
	Healthy Ind.	1	28	0	2.37	0.0934	2.20	2.59
		2	26	2	2.37	0.0891	2.20	2.59
		3	26	2	2.38	0.0838	2.20	2.58
SUPERIORTEMPORAL THICKNESS	rec MDD	1	18	0	2.88	0.1558	2.64	3.20
		2	17	1	2.85	0.1337	2.60	3.08
		3	16	2	2.87	0.1547	2.63	3.15

Descriptives

	Participant Group	Time Point	N	Missing	Mean	SD	Minimum	Maximum
	Healthy Ind.	1	28	0	2.99	0.1452	2.59	3.19
		2	26	2	2.99	0.1414	2.57	3.19
		3	26	2	3.02	0.1136	2.76	3.21
SUPRAMARGINAL THICKNESS	rec MDD	1	18	0	2.63	0.1123	2.34	2.80
		2	17	1	2.61	0.0940	2.41	2.75
		3	16	2	2.63	0.1167	2.41	2.82
	Healthy Ind.	1	28	0	2.73	0.0938	2.49	2.89
		2	26	2	2.73	0.0935	2.52	2.90
		3	26	2	2.74	0.0898	2.52	2.91
FRONTALPOLE THICKNESS	rec MDD	1	18	0	2.86	0.1582	2.63	3.19
		2	17	1	2.83	0.1299	2.57	3.12
		3	16	2	2.89	0.1654	2.67	3.30
	Healthy Ind.	1	28	0	2.95	0.2300	2.49	3.38
		2	26	2	2.93	0.1916	2.55	3.21
		3	26	2	2.97	0.2155	2.51	3.35
TEMPORALPOLE THICKNESS	rec MDD	1	18	0	3.76	0.2069	3.19	4.11
		2	17	1	3.78	0.1848	3.41	4.14
		3	16	2	3.77	0.1938	3.42	4.15
	Healthy Ind.	1	28	0	3.85	0.2266	3.40	4.23
		2	26	2	3.84	0.2391	3.45	4.26
		3	26	2	3.84	0.2636	3.32	4.28
TRANSVERSETEMPORAL THICKNESS	rec MDD	1	18	0	2.43	0.2152	2.06	2.78
		2	17	1	2.43	0.1891	2.06	2.69
		3	16	2	2.45	0.2283	2.08	2.83
	Healthy Ind.	1	28	0	2.61	0.1935	2.23	3.14
		2	26	2	2.61	0.1777	2.37	3.21
		3	26	2	2.62	0.1883	2.35	3.12
INSULA THICKNESS	rec MDD	1	18	0	3.08	0.1503	2.79	3.29
		2	17	1	3.07	0.1361	2.82	3.32
		3	16	2	3.09	0.1503	2.80	3.34
	Healthy Ind.	1	28	0	3.18	0.1393	2.91	3.48
		2	26	2	3.16	0.1257	2.89	3.40
		3	26	2	3.18	0.1433	2.92	3.55

Descriptives - Subcortical Volumes and Cerebellum

	Participant Group	Time Point	N	Missing	Mean	SD	Minimum	Maximum
Thalamus	rec MDD	1	18	0	5360	385.7	4706	6288
		2	18	0	5295	400.8	4748	6356
		3	17	1	5334	404.1	4787	6327
	Healthy Ind.	1	28	0	5421	449.9	4777	6727
		2	26	2	5415	467.2	4707	6741
		3	26	2	5416	460.7	4697	6729
Caudate	rec MDD	1	18	0	2457	270.6	2017	2991
		2	18	0	2446	249.1	2018	2962
		3	17	1	2431	261.3	2038	2986
	Healthy Ind.	1	28	0	2531	216.6	1879	2968
		2	26	2	2528	226.1	1891	2924
		3	26	2	2503	226.5	1884	2964
Putamen	rec MDD	1	18	0	3509	324.5	3132	4317
		2	18	0	3477	344.4	3063	4355
		3	17	1	3502	315.5	3116	4242
	Healthy Ind.	1	28	0	3438	326.4	2802	4393
		2	26	2	3411	319.6	2802	4428
		3	26	2	3447	330.7	2788	4457
Pallidum	rec MDD	1	18	0	1297	148.7	1070	1596
		2	18	0	1313	138.0	1075	1559
		3	17	1	1294	143.6	1059	1575
	Healthy Ind.	1	28	0	1313	109.8	1094	1501
		2	26	2	1307	102.0	1100	1495
		3	26	2	1306	107.2	1074	1492
Hippocampus	rec MDD	1	18	0	3044	253.6	2674	3575
		2	18	0	3048	238.6	2676	3480
		3	17	1	3060	245.1	2672	3527
	Healthy Ind.	1	28	0	3080	250.2	2704	3500
		2	26	2	3073	255.6	2700	3552
		3	26	2	3097	240.0	2747	3534
Amygdala	rec MDD	1	18	0	1142	108.2	845	1272
		2	18	0	1149	106.5	848	1268
		3	17	1	1149	101.2	851	1287
	Healthy Ind.	1	28	0	1141	105.3	931	1370
		2	26	2	1136	102.4	918	1332
		3	26	2	1158	94.3	1013	1354
Accumbens	rec MDD	1	18	0	369	81.8	257	551
		2	18	0	364	72.9	275	544
		3	17	1	372	76.4	254	548
	Healthy Ind.	1	28	0	374	37.5	288	442
		2	26	2	372	39.4	271	444
		3	26	2	376	43.6	267	461
Cerebellum	rec MDD	1	18	0	39928	3002.4	35389	47050
		2	18	0	39667	3050.7	34862	47131
		3	17	1	40041	3060.2	34880	46761
	Healthy Ind.	1	28	0	40978	3122.9	35474	49750
		2	26	2	41079	3315.9	35462	50006
		3	26	2	41078	3074.9	35635	49641
